# Geopolymer-Based Materials for the Removal of Ibuprofen: A Preliminary Study

**DOI:** 10.3390/molecules29102210

**Published:** 2024-05-08

**Authors:** Rosanna Paparo, Martino Di Serio, Giuseppina Roviello, Claudio Ferone, Marco Trifuoggi, Vincenzo Russo, Oreste Tarallo

**Affiliations:** 1 Department of Chemical Sciences, University of Naples Federico II, 80126 Naples, Italy; rosanna.paparo@unina.it (R.P.); diserio@unina.it (M.D.S.); marco.trifuoggi@unina.it (M.T.); 2 Department of Engineering, University of Naples ‘Parthenope’, Centro Direzionale, Isola C4, 80143 Napoli, Italy; giuseppina.roviello@uniparthenope.it (G.R.); claudio.ferone@uniparthenope.it (C.F.); 3 INSTM Research Group Napoli Parthenope, National Consortium for Science and Technology of Materials, Via G. Giusti, 9, 50121 Firenze, Italy

**Keywords:** adsorption, emerging contaminants, geopolymers, hybrid materials, ibuprofen, sorbents

## Abstract

Every year, new compounds contained in consumer products, such as detergents, paints, products for personal hygiene, and drugs for human and veterinary use, are identified in wastewater and are added to the list of molecules that need monitoring. These compounds are indicated with the term emerging contaminants (or Contaminants of Emerging Concern, CECs) since they are potentially dangerous for the environment and human health. To date, among the most widely used methodologies for the removal of CECs from the aquatic environment, adsorption processes play a role of primary importance, as they have proven to be characterized by high removal efficiency, low operating and management costs, and an absence of undesirable by-products. In this paper, the adsorption of ibuprofen (IBU), a nonsteroidal anti-inflammatory drug widely used for treating inflammation or pain, was performed for the first time using two different types of geopolymer-based materials, i.e., a metakaolin-based (GMK) and an organic–inorganic hybrid (GMK-S) geopolymer. The proposed adsorbing matrices are characterized by a low environmental footprint and have been easily obtained as powders or as highly porous filters by direct foaming operated directly into the adsorption column. Preliminary results demonstrated that these materials can be effectively used for the removal of ibuprofen from contaminated water (showing a concentration decrease of IBU up to about 29% in batch, while an IBU removal percentage of about 90% has been reached in continuous), thus suggesting their potential practical application.

## 1. Introduction

**Scheme 1:**
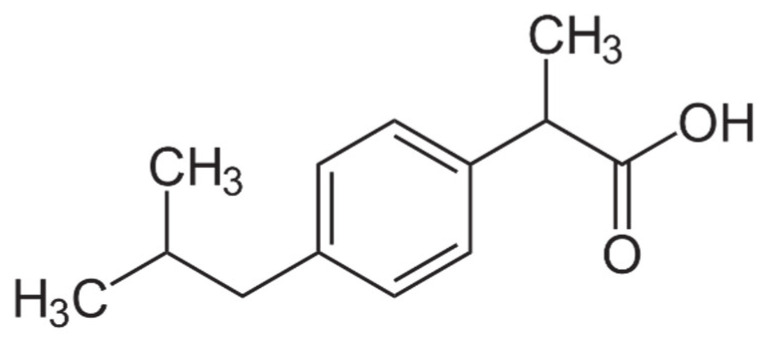
Molecular formula of ibuprofen, C_13_H_18_O_2_, CAS No.: 15687-27-1.

Safe management of hazardous compounds contained in solid and liquid wastes constitutes an important environmental challenge. In recent years, compounds deriving from drugs for human or veterinary use, hormones, and chemicals contained in commonly used products, such as personal hygiene cleaners, disinfectants, and cosmetics, have been identified in civil and industrial wastewater [1]. Since current treatment plants are not yet able to remove these substances effectively [2], these compounds are found, even in high concentrations, in purified sewage water, in surface waters of rivers and lakes, and even in drinking water: for example, the presence of ibuprofen in water can be quantified in the range 0.03–1.2 µg/L in the USA and around 3.5 µg/L in Germany [3] while in Italy it ranges from 0.1 to 1 µg/L [4]. Furthermore, following oxidative processes or partial degradation processes, these molecules often give rise to other compounds, so their removal is very complex and generally not feasible by conventional microbial degradation techniques [5]. These pollutants are called “emerging contaminants”, not as “new” substances, but because their presence in the environment and the resulting (eco)toxicity have only recently emerged [6]. For this reason, even if they could present a significant risk that requires regulation, they are very often not yet included in routine environmental monitoring programs. Ibuprofen (IBU, Table 1, Scheme 1) is one of the most commonly used non-steroidal anti-inflammatory drugs (NSAIDs) in the world and is recognized as an emerging contaminant. Several kilotons of IBU are produced each year [7]. After its use, IBU is excreted by patients unmetabolized or as metabolites deriving from human biodegradation to be emitted into raw sewage, which may or may not be treated [8]. The existence of these pharmaceuticals in the aqua environment (effluents, rivers, tributaries, and public tap water), even at low concentrations (ngL^−1^–μgL^−1^), could represent a potential danger to public health. Different mitigation methods (physical, chemical, and biological) have been studied for IBU removal and, among them, the adsorption process allows for the use of wastes and other cheap, sustainable, and renewable materials [9].

In the last years, several methods, such as adsorption, membrane filtration, ozonation, and advanced oxidation processes, have been implemented for the removal of emerging contaminants [11]. Among these, adsorption is the technique of choice, as it is characterized by ease of implementation, simple equipment that is easily coupled, and low operating costs [12]. Moreover, in some cases, the extension of the adsorption as a continuous operation is not always straightforward, and specific experimental data need to be collected [13].

To date, many materials have been developed as adsorbents (activated carbon, zeolites, metal–organic frameworks, or ion exchange resin) [14,15,16,17,18]. However, some drawbacks, such as poor adsorption performances, small surface area, difficult regeneration, and non-competitive cost, can limit their application in the extensive treatment of wastewater. Thus, the development and use in the treatment of wastewater of new adsorbents deriving from raw materials characterized by a low environmental impact could be helpful both to the environment and to the economy since these materials will present a very low production cost and, being obtained from industrial wastes, can contribute to solving the waste disposal problem.

Among this class of materials, geopolymer-based adsorbents can be considered very promising, thanks to their low environmental footprint, low cost, and interesting properties such as good chemical resistance and mechanical properties, long-term durability, and ability to be easily recycled [19,20,21]. Geopolymers are amorphous aluminosilicates containing alkaline metals that are characterized by a 3D structure similar to tectosilicates (a 3D-network formed by tetrahedra having a Si atom in the center and O atoms in common with the neighboring tetrahedra) in which, similarly to what is observed in clays, a portion of the Si atoms are replaced by Al atoms [22,23]. Their general formula is M_n_[–Si–O_2_]*_z_*[–Al–O]*_n_*•wH_2_O, where *M* is an alkaline metal and *z* and *n* are variable numbers indicating the SiO_2_/Al_2_O_3_ molar ratio. Geopolymers can be obtained by reacting a powdered precursor with a high content of amorphous aluminosilicates, with a strong alkaline solution in mild conditions [23]. Geopolymers are considered to have a low environmental impact and low cost as they can be prepared from waste raw materials and/or industrial by-products (fly and bottom ash, blast furnace slag, water purification sludge, etc.) that would otherwise be disposed of, or by starting from clays (e.g., metakaolin).

The ability of geopolymer-based materials to adsorb various chemical species, mainly metal cations, is well described in the literature [24,25]. This property derives from the intrinsically polyanionic porous structure of the geopolymer, similar in nature to that of zeolites, which is characterized by the presence of irregular nanopores of 3–10 nm in diameter [26]. Actually, zeolite-rich phases can sometimes be found in geopolymers [27]. Since zeolites have high microporosity and geopolymers have mesopores [23], zeolite–geopolymer hybrid bulk materials can have a wide pore size distribution. By adding a foaming agent to the geopolymeric mixture before hardening, foams can also be obtained [28,29]. This method (direct foaming) consists of generating bubbles in the suspension by adding a blowing agent capable of reacting in situ with the water molecules, developing gas [30]. With this procedure, foams with a wide density range characterized by both closed and open cells are obtained [28]. An increase in the concentration of the foaming agent causes an increase in total porosity and an increase in the size of the pores.

The main agents used for direct foaming are the following: (i) hydrogen peroxide, which decomposes to water and oxygen through an exothermic reaction 2H_2_O_2_ = 2H_2_O + O_2_; (ii) metal powders such as aluminum and silicon which, in alkaline solutions, release gaseous hydrogen; (iii) fillers containing impurities capable of generating porosity, such as “silica fume” or silicon carbide, which, depending on the free silicon contained in them, release hydrogen.

In this framework, geopolymer-based foams with a controlled micro- and macro-porosity and high surface area have been described by some of us by using organic–inorganic hybrids obtained, in turn, by a co-reticulation process in the geopolymer slurry of oligomeric polydimethylsiloxanes [31,32].

To date, even if cationic adsorption in geopolymers has been thoroughly studied, anionic entrapment is a less studied topic [33] and, to the best of our knowledge, no data have been reported on the absorption of emerging pollutants [34,35,36], in particular with hybrid organic–inorganic geopolymer-based materials.

This work proposes, for the first time, the use of geopolymers and geopolymer-based hybrid materials in the creation of adsorbing artifacts for the removal of emerging organic pollutants. In particular, the study of the adsorption of ibuprofen on neat geopolymeric substrate and on a new geopolymer–polydimethylsiloxane hybrid substrate is reported. Moreover, for the first time, the adsorbing substrate materials were made in situ in steel columns which were used to study the adsorption process and kinetics. Batch and column adsorption experiments showed that both the materials are effective at adsorbing ibuprofen and, furthermore, through desorption tests, it has been shown that the adsorbed pollutant is not appreciably released into the aqueous medium.

## 2. Results and Discussion

### 2.1. Synthesis and Characterization of the Absorbing Materials

#### 2.1.1. Preparation and Characterization of the Absorbing Foams

The adsorbing columns were produced by direct foaming of the geopolymeric materials, first in long transparent organic glass test tubes (internal diameter 1.5 cm, length 15 cm), to verify the ability of the material to expand uniformly and homogeneously occupy the column. These preliminary foaming tests into transparent tubes were necessary because the different formulations used, by virtue of the different chemical compositions and desired degree of foaming, contained different quantities of foaming agent, which made the final volumes of the various mixtures vary compared to the starting volumes.

**Figure 1:**
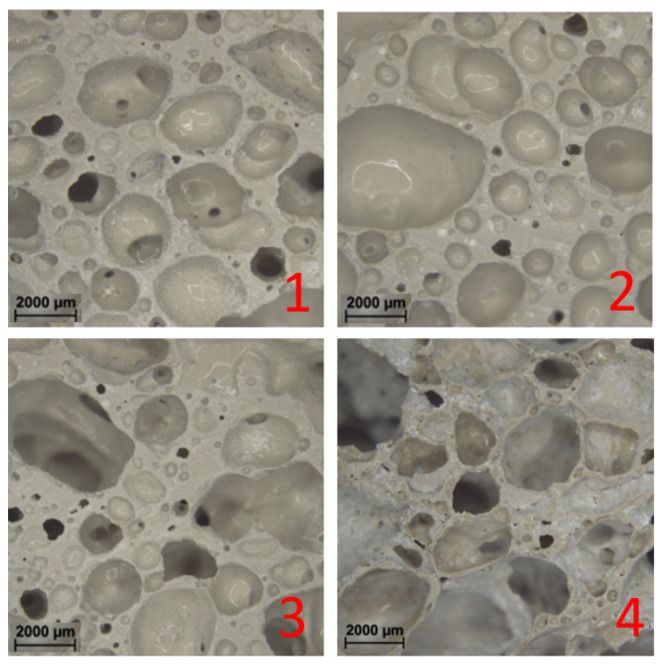
Optical images of longitudinal sections of in situ foamed samples obtained by direct foaming into transparent glass cylinders and curing of different geopolymeric samples: (**1**) GMK10, (**2**) GMK20, (**3**) GMK30, (**4**) GMK-S55.

In accordance with what has already been reported in previous papers by some of us on geopolymer samples with similar compositions [31], we noticed that the neat geopolymer slurries (without DMS oligomers) loaded with a foaming agent content ranging from 0.015% by weight to 0.05% (i.e., samples GMK015 to GMK05) showed little increase in volume and did not produce homogeneous foams since the viscosity of the mixture was very low. At variance, by using a larger amount of foaming agent (≥10 wt.%), a good foaming level was obtained. As far as the organic–inorganic hybrid samples, in which also some DMS oligomers were added in the geopolymer slurry (see for example, GMK-S55 mixture), the mixture doubled in volume in a few minutes. In order to assess the efficiency of the foaming process and the shape and distribution of the pores, the foam samples prepared in test tubes were sectioned longitudinally and subjected to a morphological analysis using optical and scanning electron microscopy. Figure 1 shows optical images of different formulations of both the geopolymer and geopolymer hybrid materials with different percentages of the foaming agent.

In all cases, the foaming was rather homogeneous, with pores ranging in size from a few microns to up to 2 mm uniformly distributed throughout the sample. As expected, the pores showed larger dimensions as the amount of foaming agent increased. Examining the images, it becomes evident that, as the foaming agent content increases, the porosity of the samples changes from closed to open. Specifically, we can observe that samples obtained with low percentages of foaming agent (GMK10 and GMK20, shown in parts 1 and 2 of Figure 1, respectively) show pores that appear closed, while samples GMK30 and GMK-S55, obtained, respectively, with 0.30 wt% and 0.55 wt% of Si in respect to the total mass of the geopolymer slurry (parts 3 and 4 of Figure 1, respectively), exhibit a continuous porosity (open porosity). For this reason, as discussed in the following sections, adsorption tests in continuous were performed on sample GMK-S55, in order to minimize pressure losses due to the continuous flow of pollutants through the column.

**Figure 2:**
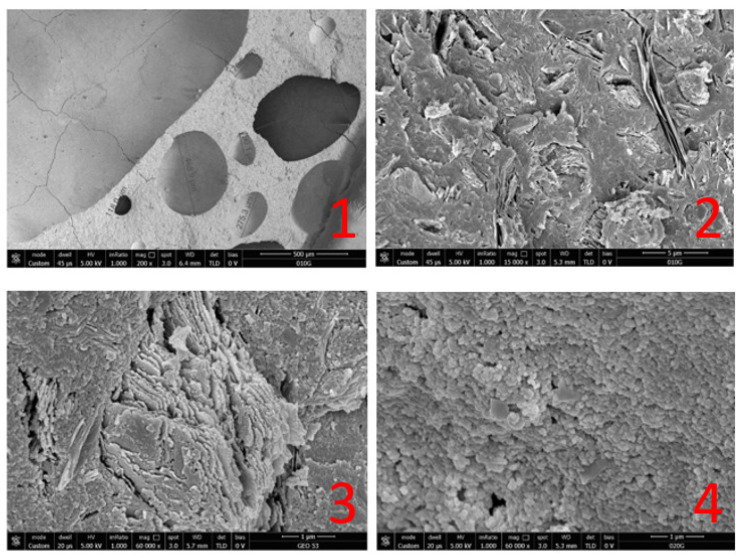
SEM micrographs of fresh fracture surfaces of the samples GMK20 at different magnifications: (**1**) 200×, (**2**) 15,000×; (**3**) and (**4**) 60,000×.

A more detailed morphological characterization of the foams was performed with the aid of a scanning electron microscope (SEM). For example, regarding the neat geopolymer sample GMK20 (Figure 2), the pores are separated by homogeneous walls which endow the foamed material with good mechanical resistance. Micro-cracks can also be observed, probably due to the stress suffered by the material when it was extracted from the test tubes and subsequently sectioned. At higher magnification (parts 2 and 3 of Figure 2), hexagonal lamellae can be observed, indicating the presence of unreacted kaolinite clay. Furthermore, in accordance with the literature [22], it is possible to notice that the geopolymer structure is made of nanometric spheres (with a diameter varying from about 20 to 60 nm) due to the gelling process and loss of water that occurs during the geopolymerization reaction. [17]. This phenomenon also justifies the presence of diffused nanovoids (with an average diameter of less than 20 nm) which make the structure of the material intrinsically porous and, therefore, potentially suitable for use as an adsorbent material.

**Figure 3:**
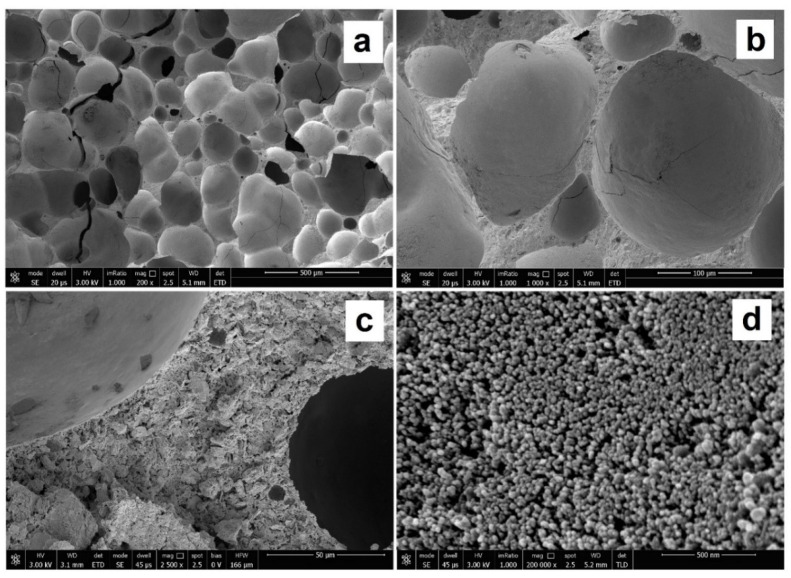
SEM micrographs at 200 (**a**), 1000 (**b**), 2500 (**c**), and 200,000 (**d**) magnification of freshly fractured surfaces of the GMK-S55 sample.

In Figure 3, SEM micrographs of the GMK-S55 hybrid sample at different magnifications are reported. Consistently with the optical images shown above, this sample shows a fairly regular distribution of pores due to the evolution of hydrogen during the foaming process (Figure 3a,b). A more in-depth analysis of the pore walls (Figure 3c) shows that the sample has a continuous and uniform structure in which no segregation phenomenon of the organic phase (i.e., dimethyl siloxane) with respect to the inorganic one (geopolymer) is observable, even at the nanometer level. This is attributable to the formation of a three-dimensional and interpenetrating network in which the inorganic (geopolymer) phase and the polysiloxane component are able to interact strongly by forming chemical bonds between the aluminosilicate and siloxane units [31]. Additionally, in this case, at very high magnification, the presence of a nodular morphology is evident, derived from the formation of the gel in the early stages of polycondensation reaction, [22] with an average diameter of nodules of about 40–50 nm (Figure 3d).

#### 2.1.2. X-ray Diffraction

**Figure 4:**
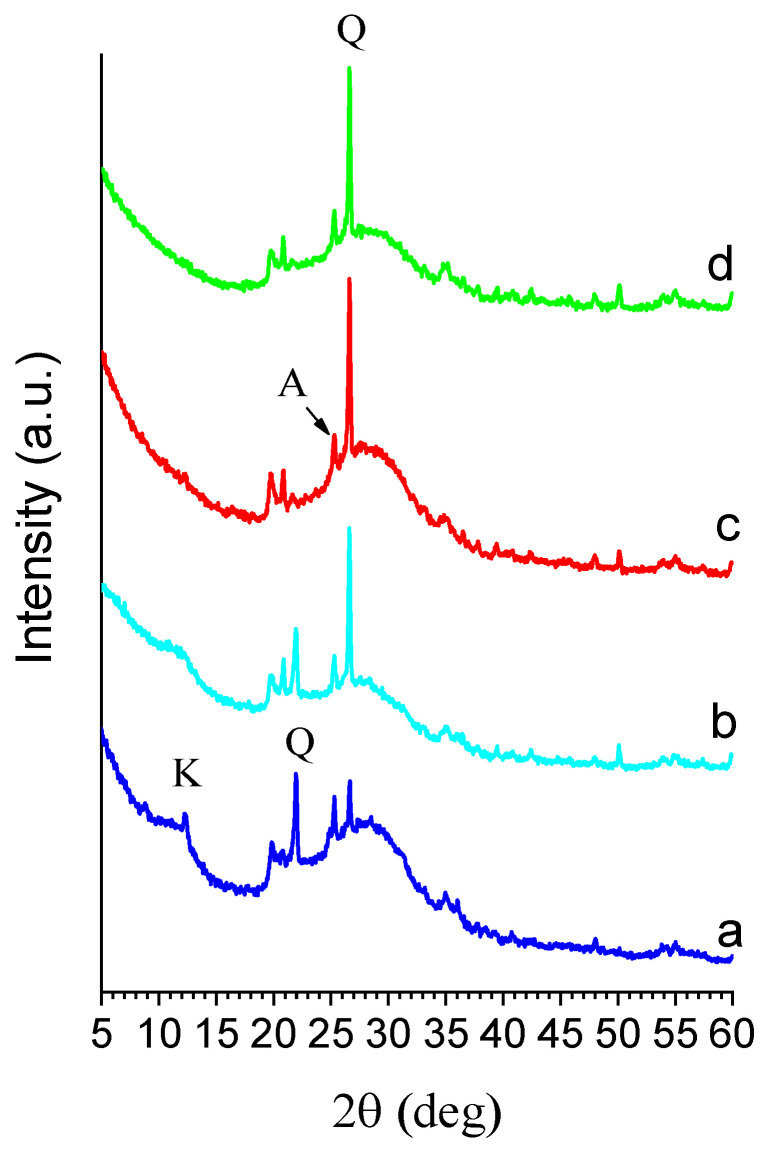
Diffraction profiles of samples GMK-S55 (**a**), GMK-S50 (**b**), GMK20 (**c**), and GMK30 (**d**). Some characteristic diffraction peaks of crystalline phases already present in the metakaolin precursor are indicated: kaolinite (K), anatase (A), and quartz (Q).

The GMK and GMK-S samples were also characterized by wide angle X-ray diffraction. Some representative diffraction patterns are reported in Figure 4. As can be observed, in agreement with the structural characterization of similar samples reported in the literature, [24,30,31,32], all the diffraction patterns are characterized by a rather wide halo centered at 2θ = 27° on which the characteristic diffraction peaks of kaolinite, anatase, and quartz (already present in the starting metakaolin from which the geopolymer samples have been synthetized) are evident.

#### 2.1.3. Zeta Potential Analysis

**Figure 5:**
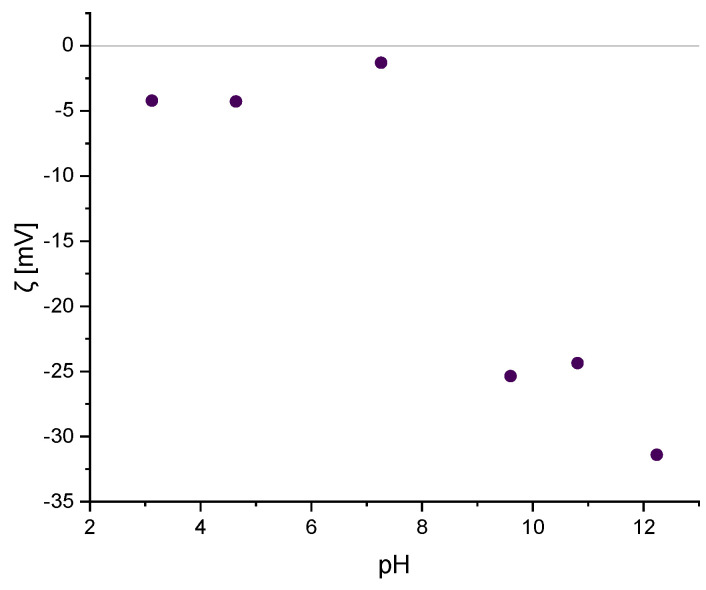
Zeta potential values as obtained by changing pH of the GMK20 geopolymer.

Zeta potential analysis of the GMK20 geopolymer sample performed at different pH values and using a refraction index equal to 2.3 is reported in Figure 5. As is apparent, in the studied pH interval (from pH = 3 to pH = 13) the adsorbent’s zeta potential is always negative.

#### 2.1.4. FT-IR Spectra

**Figure 6:**
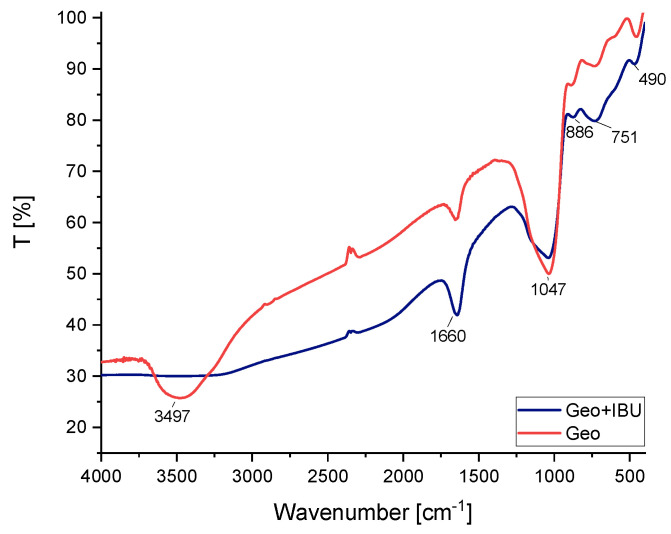
FT-IR spectra of pristine GMK20 geopolymer (red) and after saturated IBU (ibuprofen) addition (blue).

Figure 6 shows the FT-IR spectra obtained for the GMK20 geopolymer sample before (pristine) and after ibuprofen adsorption. The peaks in the geopolymer’s FT-IR spectra at 3497–1660 cm^−1^ are attributable to –O–H stretching and to the deformation and H–O–H hydrogen-binding from the water molecules. The peak at 1047 cm^−1^ is likely due to Si–O–Si deformation and the stretching vibration of the groups Si–O and asymmetric Al–O–Al/Si–O–Si stretching. The signal at 751 cm^−1^ represents the bending vibration Si–O–Si, while at 490 cm^−1^ there is a characteristic band of Al–O/Si–O bending vibration. After the adsorption of IBU onto the geopolymer the intensity of the peaks slightly decreased because of the interactions between the functional groups of the adsorbent material and ibuprofen and the shielding effect of the adsorbed molecules, meaning that functional groups on the surface were involved in the adsorption of ibuprofen [37].

#### 2.1.5. Thermogravimetric Analysis

**Figure 7:**
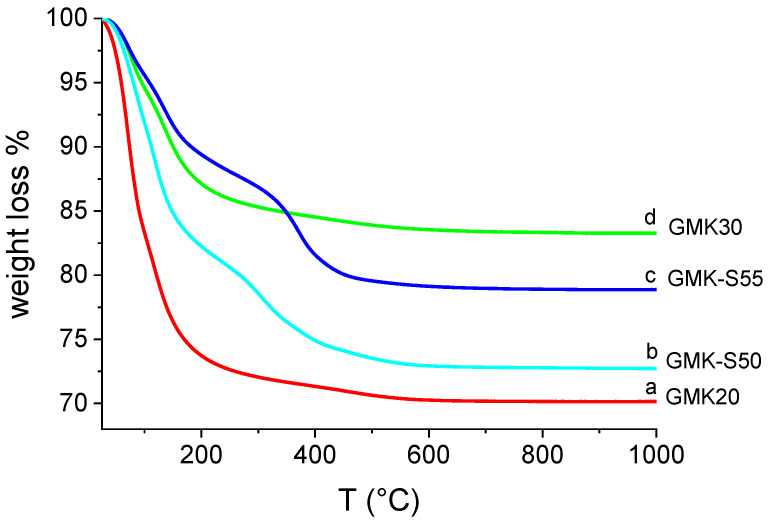
Thermogravimetric analysis of the samples: GMK20 (**a**), GMK-S50 (**b**), GMK-S55 (**c**) and GMK30 (**d**).

Samples were finally characterized by a thermogravimetric analysis run in air to study their thermal stability. As can be seen from Figure 7, at a temperature of 200 °C the samples GMK20 and GMK30 show a weight loss of about 15% and about 30%, respectively. This loss is likely attributable to the removal of free water present in the samples (up to T = 100 °C) or water absorbed on the surface of the material and, at higher temperatures (up to about 200 °C), water present in the nanopores or bound [38,39,40], which is present to a greater extent in sample GMK30 than in sample GMK20 due to the different compositions of the starting mixtures (see Table 4 in Section 3.2.4).

An analogous behavior is observed for the hybrid samples GMK-S50 and GMK-S55, which show, in the temperature range between 350 and 450 °C, a further loss in weight due to the decomposition of the polydimethylsiloxane component present in the sample structure [41].

### 2.2. Adsorption Tests

Adsorption tests performed in batch showed a low affinity between the geopolymers and ibuprofen (IBU). The solution of IBU with a concentration of 15 mg/L had a pH value of 4.5, so, the IBU coexists in a neutral and anionic form. This could be explained by the fact that geopolymers are composed of polyanionic 3D frameworks, made up of alternating SiO_4_^4−^ and AlO_4_^5−^ tetrahedra, that, at pH = 4.5, are positively charged according to a Z potential analysis reported in the literature [42].

**Figure 8:**
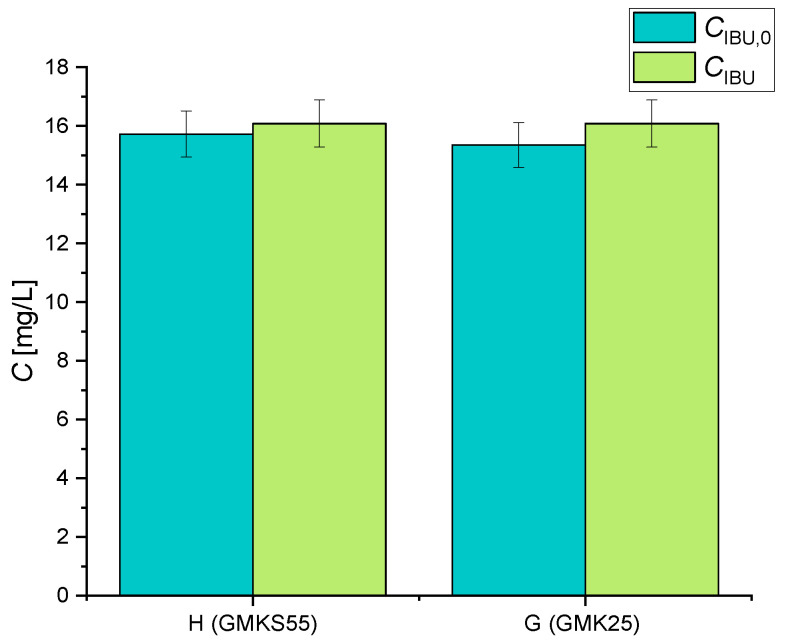
IBU concentration [mg/L] values obtained by performing adsorption tests, t: 1440 min, T = 303 K. A total of 0.112 g of each geopolymer has been used in the tests.

Figure 8 shows the concentrations before and after adsorption was performed at the pristine pH of the solution (4.5), with 0.112g of H (GMK-S55) and 0.112g of G (GMK25) geopolymers at room temperature. As is visible, no adsorption occurs after 120 min. No adsorption was measured in the whole experimental time set from 0 to 120 min (condition reported in Table 4).

**Figure 9:**
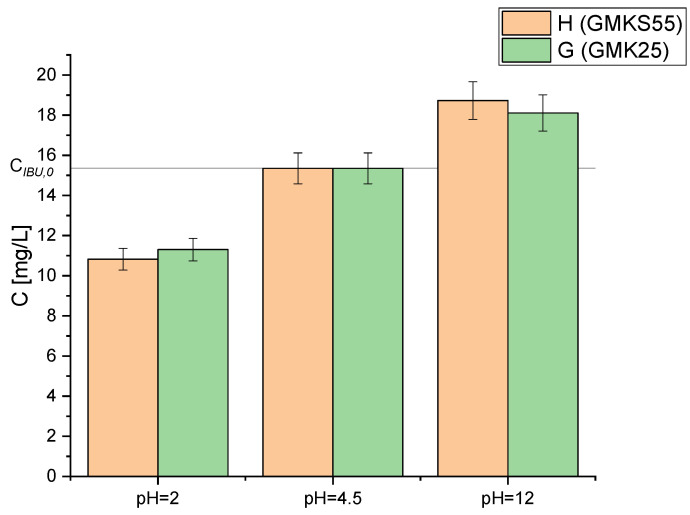
IBU concentration after the adsorption tests at different pH (2, 4.5, and 12) for both H and G geopolymers after 1440 min at T = 303K. The amount of used geopolymer was 0.008 g.

Different adsorption tests were performed by changing the initial pH of the solution prepared, both in a basic condition with a pH = 12 (using NaOH) and in an acid condition with a pH = 2 (using HCl), at room temperature, and using 0.008 g of each geopolymer. Figure 9 shows an increase in the adsorption of IBU by geopolymers at pH = 2, and, in fact, IBU concentration decrease by 26.38% (*q_t_* = 5.66 mg/g) and by 29.51% (*q_t_* = 5.06 mg/g) for G and H geopolymers, respectively.

**Figure 10:**
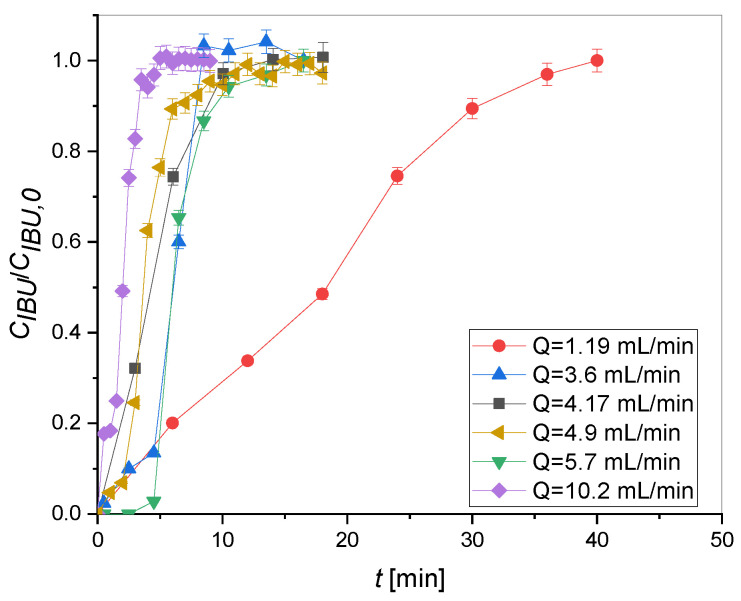
Comparison of kinetic tests at different flow rates carried out using column H.

As far as the continuous adsorption tests, six adsorption tests were carried out with GMK-S55 (column H) at different flow rates (Q = 1.19, 3.6, 4.17, 4.9, 5.7, 10.2 mL/min) (Figure 10). At low flow rates, the system reached saturation in a very long time, with the shape of the curve deviating from the ideality of fluid dynamics. As the volumetric flow rate increased, the dimensionless concentration profiles varied, resulting in behaviors closer to ideality as the data dispersed along a sigmoid. In general, the inflection point decreases as the volumetric flow rate increases, indicative of a shorter residence time of the fluid elements within the adsorption column.

**Figure 11:**
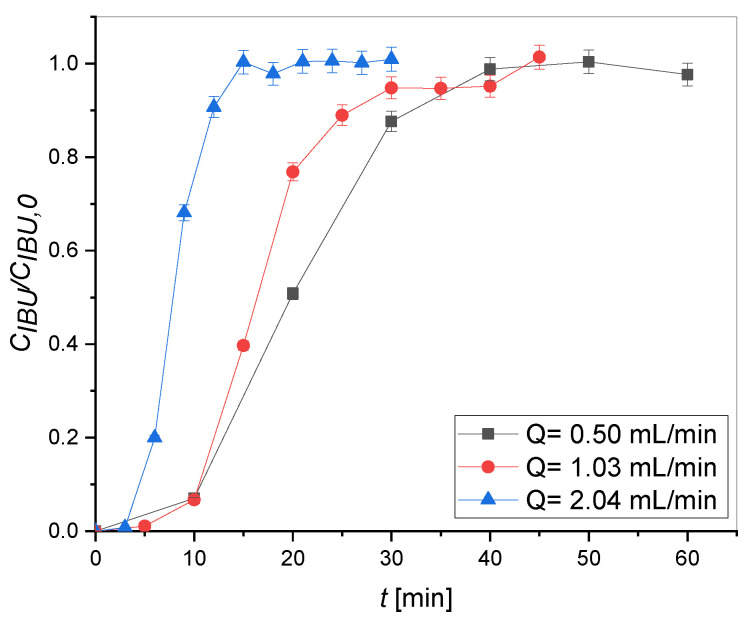
Comparison graph of kinetic tests at different flow rates performed on column G.

The data obtained for GMK25 (column G) also show that the shape of the curves approximates a sigmoid characterized by a steeper slope for higher flow rates, indicative of fluid dynamics closer to ideality (Figure 11). Again, the characteristic time of the inflection point decreases as the volumetric flow rate increases, indicative of a shorter residence time of the fluid elements within the adsorption column.

**Figure 12:**
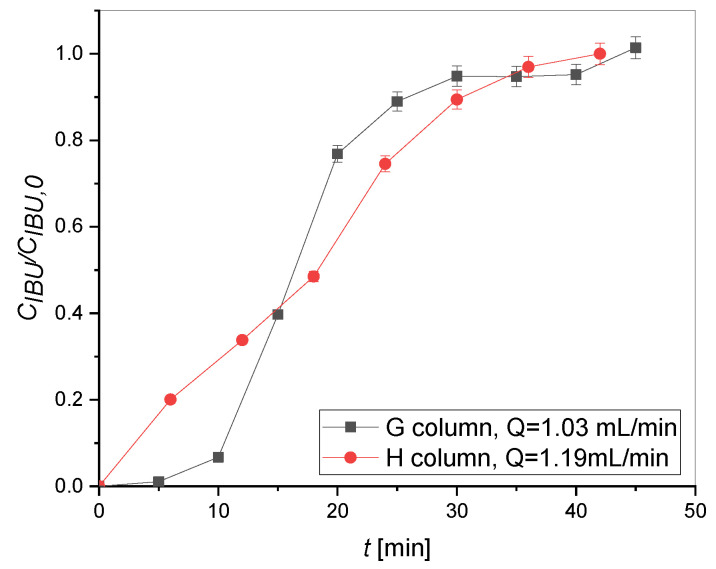
Comparison between the adsorption breakthrough curves obtained for sorbents G and H.

Figure 12 shows a comparison of the results of the kinetic tests conducted with the two different columns at the same volumetric flow rate. It was found that column G exhibited better fluid dynamics than column H, as the data in the former case are dispersed around a sparsely spanned sigmoid.

**Figure 13:**
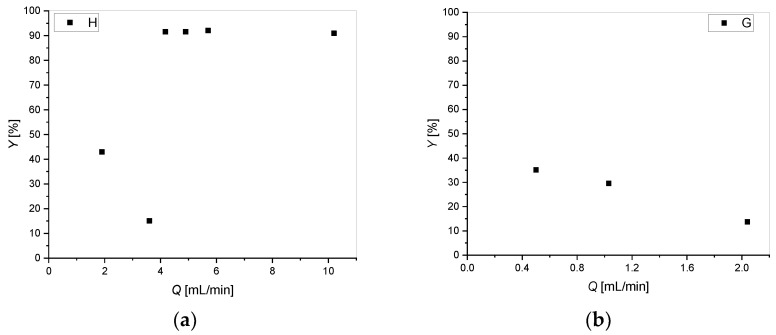
Absorption percentage for columns H (**a**) and G (**b**).

To understand the IBU adsorption performances obtained using the H and G columns, a solution of ibuprofen (*C*_0_ = 10 mg/L) was pumped into the columns at different flow rates. The percentage of IBU removal Y was calculated using Equation (4). Figure 13a shows that the value of the adsorbed amount of ibuprofen increases as the flow rate increases until it reaches a maximum flow rate of 5.70 mL/min (Table 2).

The behavior for column G is less predictable as the fluid dynamics are non-ideal (Figure 13b). In this case, by increasing the flow rate, a decrease in the adsorption percentage is observed, probably due to the lower residence time, thus, molecules are washing out the column.

### 2.3. Adsorption Mechanism

Figure 9 shows that pH 2 will induce the maximum attraction between the adsorbent (negatively charged) and the acidic drug not charged at this pH value and will, therefore, promote higher IBU removal efficiency. In the case of ibuprofen (pKa = 4.91), the ibuprofen exists as a neutral species at pH < pKa, coexists as a neutral and anionic species at pH ≈ pKa, and exists as an anion species at pH > pKa [43]. Ibuprofen becomes negatively charged as the pH rises above 4. In fact, at this value of pH, the geopolymer’s surface has a less negative charge because of the protonation of aluminol and silanol species on the surface (i.e., Al–OH + H^+^ ⇌ Al–OH_2_^+^) [42] while IBU is in its neutral form. The interaction of the adsorbent–adsorbate rises due to both hydrogen bonding among silanol and aluminol groups of the geopolymer with carboxylic groups (–COOH) of the IBU and ligand-exchange adsorption. Anionic carboxyl groups of ibuprofen molecules were attracted to the Bronsted acid sites of Si–(OH)+ –Al through an electrostatic interaction.

### 2.4. Adsorption/Desorption Tests

As the experiments conducted using column H showed a higher IBU remotion percentage [Y%] than the ones performed using column G (Figure 11), several adsorption and desorption experiments were conducted to also check the performance of the column in wash-out mode, thus, pre-saturating the column and then feeding water at the entrance of the pipe and measuring the desorption of the pollutant at the outlet of the pipe.

Two independent series of adsorption experiments were carried out, one with a feed flow of 2.7 mL/min, and one with a flow of 5.4 mL/min. Periodic sampling was then performed for each series (for the 2.7 mL/min flow every 2 min and for the 5.4 mL/min flow every minute) so that volume fractions of about 5 mL were collected. After making sure that we had reached the saturation concentration of ibuprofen (corresponding to the feed concentration), we then fed the column with distilled water to study the desorption process. Specifically, for a feed flow rate of 5.4 mL/min, a value of breakpoint of 5 min was obtained, while, for a flow rate of 2.7 mL/min, the value became about 2 min. Doubling the flow rate resulted in an increase in the breakpoint time, which is the time useful for adsorption. As the flow rate increases, the time the pollutant molecules have to interact with the surface of the solid decreases; therefore, the breakpoint time tends to decrease. Since, in our case, this time increases, the reason is to be found in the presence of fluid–solid diffusive phenomena, which are responsible for the lower efficiency of the column and tend to become more negligible when the flow rate increases.

**Figure 14:**
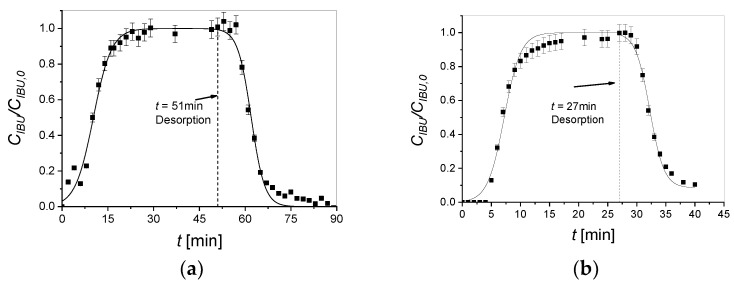
Adsorption/desorption curves for column H imposing volumetric flow rates of 2.7 (**a**) and 5.4 (**b**) mL/min.

Figure 14a,b shows the adsorption/desorption curve for the test performed with a flow rate of 2.7 mL/min and that for the test performed with a flow rate of 5.4 mL/min, respectively.

From these graphs, it can be seen that the concentration of ibuprofen is increasing according to a sigmoidal trend in adsorption tests and decreasing for desorption tests. During an adsorption test, there is an initial time when no ibuprofen is released at the column outlet appreciably. During this time, the bed adsorbs ibuprofen. A breakpoint time can be defined as the time for a fractional amount of pollutant equal to 5% to be detected at the outlet. Specifically, for a feed flow rate of 5.4 mL/min, a value of 5 min is obtained, while, for a flow rate of 2.7 mL/min, the value becomes about 2 min. That is, doubling the flow rate results in an increase in the breakpoint time, which is the time useful for adsorption. This result is not trivial because, generally, as the flow rate increases the time the pollutant molecules have to interact with the surface of the solid decreases; therefore, the breakpoint time tends to decrease. Since, in our case, this time increases, the reason is to be found in the presence of fluid–solid diffusive phenomena, which are responsible for the lower efficiency of the column and tend to become more negligible when the flow rate increases.

## 3. Materials and Methods

### 3.1. Materials

The metakaolin was kindly provided by Neuvendis s.p.a. (Milan, Italy) and commercialized under the trade name Mefisto^®^. The sodium silicate solution was supplied by Prochin Italia S.r.l (Caserta, Italy). The chemical composition of the used metakaolin and of the sodium silicate solution are reported in Table 3. Sodium hydroxide with reagent grade and silicon powder (99%, 325 mesh) were supplied by Sigma-Aldrich (Milan, Italy). Ibuprofen, (±)-2-(4-Isobutylphenyl)propanoic acid (IBU), was supplied by Alfa Aesar™ (Lancashire, Heysham) and used without further purification. Polydimethylsiloxane oligomers (DMS) Globasil AL20 mixture was provided by Globalchimica S.r.l. (Lombardore, Italy).

### 3.2. Methods

#### 3.2.1. Physical–Chemical Characterization of the Adsorbent

SEM analysis was carried out using a Phenom Pro X Microscope (Phenom-World B.V., Eindhoven, The Netherlands) on fresh fracture surfaces, after metallization with gold. The acceleration potential used was between 5 and 15 kV. The EDS analysis was conducted with a BSD detector in full mode. Optical microscopy analyses were carried out using a Zeiss Axio Imager A1 microscope (Carl Zeiss Industrielle Messtechnik GmbH, Oberkochen, Germany).

Hydrostatic weighting for apparent density and open porosity measurements was carried out employing an, OHAUS-PA213 balance (OHAUS Europe GmbH, Nänikon, Switzerland).

X-ray diffraction patterns were obtained with Ni-filtered Cu-K_α_ radiation (λ = 0.15406 nm) at room temperature (20 °C) with an automatic Rigaku powder diffractometer mod. Miniflex 600, operating in the θ/2θ Bragg-Brentano geometry. The phase recognition was carried out by using the PDF-2 2022 (International Centre for Diffraction Data^®^) database and the Rigaku PDXL2 software (ver. 1.8).

To perform zeta potential measurements, the Zetasizer Nano-ZSP (Malvern^®^, Worcestershire, UK) instrument was used, equipped with a helium–neon laser of 4 mW output power with a fixed wavelength of 633 nm (wavelength of laser red emission). Six aqueous solutions of different pH values (from 3 to 14) were prepared. Experiments were carried out at a constant temperature (25.0 ± 0.1) °C.

FT-IR spectra were obtained at a resolution of 2cm^−1^ over the range of 400–4000 cm^−1^ using FT-IR 4700LE (JASCO, Tokyo, Japan) with ATR (attenuated total reflectance). The spectra of the pristine geopolymer powdered were obtained by mixing the sample with potassium anhydrous bromide (KBr) (also used as reference material) and the mixture was pressed into a pellet prior to analysis. To identify the interaction between the geopolymer and IBU, the pellet was immersed in a drop of a saturated ibuprofen solution.

The TGA analysis of the samples was obtained with a Mettler Toledo-TGA/DSC 2 Star* System (Mettler-Toledo, Columbus, OH, USA) and was carried out in air on aliquots of approximately 15 mg of finely ground sample, with a temperature ramp ranging from 25 °C to 1000 °C and with a heating rate of 10 °C/min.

The concentration of the ibuprofen solution was checked via UV-Vis by using a UV-Vis Jasco V-550 (JASCO Europe s.r.l., Cremella, Italy) to measure the intensity of the characteristic absorption peak at 222 nm and comparing it with a previously determined calibration line (see Supplementary Materials).

#### 3.2.2. Geopolymer-Based Foams

##### Preparation of Neat Geopolymeric Mixture (GMK)

The geopolymerization reaction is based on the alkaline activation of an aluminosilicate raw material using a strongly alkaline solution [44,45]. This alkaline solution has been prepared by dissolving solid sodium hydroxide into a sodium silicate solution. The obtained solution was then allowed to equilibrate and cool for 24 h. The composition of the obtained solution can be expressed as Na_2_O:1.55 SiO_2_:12.14 H_2_O. Metakaolin was then added to the activating solution with a liquid-to-solid ratio of 1.4:1 by weight and mixed by a mechanical mixer for 10 min at 800 rpm. In Table 3, the mix composition of these geopolymer samples (GMK) is reported.

#### 3.2.3. Preparation of Geopolymer-Based Hybrid Mixture (GMK-S)

The hybrid material GMK-S was obtained by adding 10% by weight of polydimethylsiloxane oligomers to the freshly prepared geopolymer suspension [31] described in the previous paragraph, and quickly incorporated by mechanical mixing (5 min at 800 rpm). The mixture is easily workable for several hours (the complete crosslinking and hardening take place in about 5–7 h at room temperature). In Table 3, the mix composition of the GMK-S samples is reported.

#### 3.2.4. Preparation of GMK and GMK-S Foams

In order to obtain a set of samples with a different degree of porosity, a foaming agent was added to the freshly prepared neat geopolymer (GMK) and hybrid-geopolymer (GMK-S) suspensions. Toward this aim, Si powder in different wt% ratios, ranging between 0.03 and 0.55%, was added and carefully dispersed in the slurry by mixing for a further 5 min at 800 rpm. These samples are hereafter indicated as GMK*xx* and GMK-S*xx*, where *xx* refers to the decimal units of the weight percentage of silicon foaming agent added to the geopolymeric paste (e.g., GMK-S03 refers to a polysiloxane-geopolymer hybrid sample with 0.03% by weight of Si content). In Table 4, the mix compositions of the prepared samples are reported.

#### 3.2.5. Curing Treatments

As soon as prepared, the specimens were cast in cylindrical (diameter 3 cm; height 5 cm) and parallelepipedal molds (5 cm × 5 cm × 12 cm). The foamed samples used in the continuous adsorption experiments were prepared in cylindrical steel tubes and in polymethylmethacrylate tubes (inner diameter 1.5 cm; length 15 cm). Soon after casting, the specimens were cured in >95% relative humidity conditions at 60 °C for 24 h. Then they were kept at room temperature for a further 6 days in >95% relative humidity conditions and, finally, for a further 21 days in air (relative humidity 75–80%).

#### 3.2.6. Preparation of the Ibuprofen Solution

To carry out the adsorption tests it was necessary to prepare an aqueous solution of a known concentration of ibuprofen of 12 mg/L. IBU has a low solubility (0.021 mg/mL at 20 °C) in water and so, to ensure complete solubilization, the prepared solution was stirred for 24 h on a stirring plate. During the procedure, exposure to light was meticulously avoided using Al foil, since IBU is photosensitive. Ibuprofen concentration during adsorption tests was recorded following its residual concentration in the solution over time, and all the samples collected during the adsorption tests were centrifugated for 60 min to separate the supernatant from the powder. The concentration of the solution was checked via UV-Vis by measuring the intensity of the characteristic absorption peak at a wavelength of 222 nm and comparing it with a previously determined calibration line (see Figures S1 and S2).

#### 3.2.7. Adsorption Test

Batch adsorption tests were carried out by using a fixed amount (10 mg) of finely powdered matrices obtained by grounding the geopolymer samples once they were consolidated. Before their use, the adsorbing powders were sieved with a 26–106 µm sieve and carefully washed in distilled water to remove water-soluble salts (in particular NaOH) until the pH was neutral. Batch adsorption experiments were conducted by loading the geopolymer powder into 150 mL of ibuprofen aqueous solution. The suspension was stirred for 120 min to allow sufficient contact between the pollutant and the matrix. Additionally, parameters such as contact time, pH, and initial pollutant concentration were varied to evaluate their influence on the adsorption process.

Adsorption kinetic tests were carried out using a 250 mL glass jacketed three-necked flask, in which 150 mL of the ibuprofen solution with a concentration of 15 mg/L was added and stirred at 450 rpm. The system was equipped with a thermostat set at 30 °C to control the temperature for the duration of the tests. The first sample was collected as the temperature reached the set point and was named t0. After that, about 0.10 g of geopolymer was added to start the test (Table 5). Samples were withdrawal at different times (0, 10, 60, 120 min) to monitor the evolution of the concentration of the pollutants over time. A small amount of the suspension (c.a., 1 mL) was collected by a syringe and centrifuged for 60 min to remove the solid material and check the residual ibuprofen concentration in the supernatant by UV-Vis spectrophotometry.

To study the effect of pH on the adsorption process, three solutions at different pH values (2, 4.5, and 12) were prepared and each of them was put in contact with two representative samples: GMK-S55 for the hybrid samples (indicated as sample H for short) and the GMK25 sample in the case of neat geopolymers (indicated as sample G for short). In particular, a pH = 4.5 is related to the pH of the ibuprofen solution, while pH = 2 and pH = 12 values were achieved using either HCl or NaOH solutions. The studied values of pH were chosen by taking into account titrations experiments pointing out that the net charge of the GMK and GMK- S surface is equal to zero at pH = 6.1 ± 0.2, so, at lower pH values it is expected to be positively charged while at pH > 6.1 it is expected to be negatively charged. To perform the tests, glass vials were used and immersed in a water bath placed on a stirring plate to keep the temperature (303 K) under control during the experiment.

In order to calculate the amount of adsorbed IBU onto the geopolymer in batch conditions, the *q_t_* value was determined using Equation (1):

(1) $$q_{t}=\left(\frac{C_{IBU,0}-C_{IBU,0}}{w_{ADS}}\right)V$$

Regarding the continuous adsorption test, prior to their use the adsorbent columns were carefully washed by flushing with distilled water continuously by means of an HPLC pump for about 7 days, until the pH, initially very alkaline, was neutral. The water eluted from the column was then characterized by UV-Vis to ensure that no pollutants were present.

At the time of each kinetic test, the pump was calibrated by pumping distilled water into the column to be able to read the flow of the solution sent to the column. Once the average flow rate was determined, the ibuprofen solution at a known concentration (10 mg/L) was pumped into the column. The eluate was then analyzed by the UV-Vis spectrophotometer to estimate the concentration of ibuprofen present. Once the saturation concentration of ibuprofen was reached, the column was then fed with distilled water to study the desorption process.

The adsorption tests were carried out using a column containing the geopolymeric organic–inorganic matrix substrate of composition H (GMK-S55), named column H. The steel tube used has a total volume of 13.80 cm^3^ (*r* = 0.45 cm, *L* = 22 cm). A second steel tube was successively used to realize the second column with a total volume of 9.41 cm^3^ (*r* = 0.45 cm, *L* = 15cm) containing geopolymer G (GMK25), named column G. The amount of ibuprofen thus obtained is expressed in terms of mg of ibuprofen adsorbed per g of adsorbent solid, *Q_IBU_*. The amount of ibuprofen adsorbed by the fully saturated bed can be calculated by Equation (2):

(2) $$Q_{IBU}=\frac{Q/1000}{w_{ADS}}\overset{t_{E}}{\underset{0}{\int }}C\;dt$$

where *w_ADS_* [g] is the adsorbent material mass present in the column, *Q* [mL/min] is the volumetric flowrate, and *t_E_* [min] is the final time of the breakthrough curve.

The maximum ibuprofen that could be adsorbed, *Q_IBU,tot_*, can be expressed as in Equation (3):

(3) $$Q_{IBU,tot}=\frac{C_{IBU,0}\;Q\;t_{E}}{1000}$$

where *C_IBU,_*_0_ [mg/L] is the ibuprofen feed concentration.

Thus, the adsorbed percentage, *Y*, can be defined as in Equation (4):

(4) $$Y=\frac{Q_{IBU}}{Q_{IBU,tot}}100$$

## 4. Conclusions

The present paper relates to the use of foamed geopolymers and geopolymer-based hybrid materials for the removal of emerging contaminants, primarily from the aquatic environment, through the adsorption process. The adsorbent was tested in batch and continuous fixed bed column modes using an adsorbing system obtained by direct foaming into cylindrical steel columns.

In particular, in the batch system, IBU adsorption occurs only in acidic conditions (pH = 2), because the geopolymer’s surface has a positive charge because of the protonation of aluminol and silanol species on its surface (i.e., Al–OH + H^+^ ⇌ Al–OH_2_^+^) [42], while IBU is in its neutral form. The removal percentages were 26.38% and 29.51% for the G and H geopolymers, respectively. In particular, the *q_t_* value of the materials we have tested in this paper is up to 5.7 mg/g, which is in line with, or even superior to, those observed for other adsorbing materials of similar composition, such as montmorillonite, kaolinite, or bentonites, already reported in the literature [16,46]. A continuous flow system was used to indagate process scalability and two adsorption columns (G, H) were tested at different flow rates to reach an IBU removal percentage of about 90% when increasing the flow rate into the system with column H. With this column, adsorption/desorption experiments were conducted, verifying that as the flow rate increases the time the pollutant molecules have to interact with the surface of the solid decreases, leading to a corresponding decrease in the breakpoint time. Therefore, these materials could be used for the abatement of pollutants and, in particular, for the production of filters or membranes in the form of foams, powders, microspheres, and granules with different form factors.

Given the promising preliminary results obtained in this study, it will be interesting to extend it to other organic molecules constituting drugs such as ketoprofen, diclofenac, and acetylsalicylic acid; hormones, antibiotics, insecticides, herbicides, organochlorine pesticides, plasticizers, and personal care products.

## Figures and Tables

**Table 1:** Properties of ibuprofen [10].

Property	Values
IUPAC name	(±)-2-(4-Isobutylphenyl)propanoic acid
Molar mass	206.28 g/mol
Solubility in water (at 25 °C)	21 mg/L
pKa	4.91

**Table 2:** IBU removal percentage Y at different flow rates for H and G columns, respectively.

Column	Q [mL/min]	Y [%]
H	1.90	42.93
	3.60	15.08
	4.17	91.54
	4.90	91.56
	5.70	92.06
	10.20	90.94
G	0.50	35.10
	1.03	29.55
	2.04	13.72

**Table 3:** Chemical composition (weight %) of the used metakaolin (Mefisto^®^) and sodium silicate solution.

Compound	Metakaolin	Sodium Silicate Solution
SiO_2_	52.90	27.40
Al_2_O_3_	42.00	-
Na_2_O	-	8.15
K_2_O	0.90	-
TiO_2_	1.95	-
Fe_2_O_3_	1.75	-
CaO	0.25	-
MgO	0.25	-
H_2_O	-	64.45

**Table 4:** Mix composition (mass, g) and apparent density of the studied samples. Regarding Si powder, in round brackets, the wt.% with respect to the total mass of the geopolymer slurry has been reported. MK = metakaolin; SS = alkaline solution; DMS = oligomeric dimethylsiloxane mixture; Si = silicon powder. Apparent densities as determined by hydrostatic weighting are reported too.

Sample	MK (g)	SS (g)	DMS (g)	Si (g, wt.%)	Apparent Density (g cm^−3^)
GMK015	50.0	70.0	-	0.018 (0.015)	1.97
GMK02	44.8	62.8	-	0.022 (0.02)	1.98
GMK03	99.9	140.2	-	0.072 (0.03)	1.96
GMK05	100.1	140.4	-	0.121 (0.05)	1.96
GMK10	202.2	186.9	-	0.381 (0.10)	1.88
GMK15	133.7	118.2	-	0.378 (0.15)	1.85
GMK20	153.8	135.8	-	0.580 (0.20)	1.62
GMK25 (G)	176.0	155.2	-	0.830 (0.25)	1.38
GMK30	211.6	187.6	-	1.198 (0.30)	1.42
GMK-S03	45.3	63.4	12.0	0.032 (0.03)	0.701
GMK-S06	43.5	60.9	10.4	0.063 (0.06)	0.508
GMK-S30	73.4	65.2	15.6	0.418 (0.30)	0.422
GMK-S35	61.5	54.6	13.0	0.407 (0.35)	0.495
GMK-S50	115.4	102.5	24.5	1.211 (0.50)	0.408
GMK-S55 (H)	119.0	105.6	25.2	1.374 (0.55)	0.386

**Table 5:** Adsorbent amount [g], ibuprofen concentration at time zero [mg/L], time [min], and pH value used for the adsorption test.

Adsorbent	Adsorbent Amount [g]	Ibuprofen Concentration [mg/L]	Time [min]	pH
G (GMK25)	0	15.35	0	4.5
	0.104	15.78	10	4.5
	0.104	15.72	60	4.5
	0.104	16.08	120	4.5
	0	15.35	0	4.5
	0.008	11.30	1440	2
	0.008	18.11	1440	12
H (GMK-S55)	0	15.27	0	4.5
	0.112	16.45	10	4.5
	0.112	17.24	60	4.5
	0.112	16.08	120	4.5
	0	15.35	0	4.5
	0.008	10.82	1440	2
	0.008	18.73	1440	12

## Data Availability

The authors declare the availability of the data reported in this paper.

## Supplementary Materials

The following supporting information can be downloaded at: https://www.mdpi.com/article/10.3390/molecules29102210/s1, Figure S1. UV-Vis spectrum of ibuprofen with a concentration of 10mg/L recorded from 200 to 600 range of wavelength.; Figure S2. UV-VIS calibration curve for ibuprofen (IBU) solutions in water.

## List of Symbols

*C*_IBU_ [mg/L]	Ibuprofen concentration
*C*_IBU,0_ [mg/L]	Ibuprofen feed concentration
*Q* [mL/min]	Volumetric flow rate
*Q*_IBU_ [mg/g]	Mass of ibuprofen adsorbed per g of adsorbent solid
*Q*_IBU,tot_ [mg/g]	Mass of total ibuprofen adsorbed per g of adsorbent solid
*q_t_* [mg/g]	Mass of ibuprofen adsorbed per g of adsorbent solid
*t*_E_ [min]	Final time of the breakthrough curve
*V* [L]	Liquid volume
*w*_ADS_ [g]	Adsorbent material mass present in the column
*Y* [%]	IBU removal percentage
**Abbreviations**	
CECs	Contaminants of emerging concern
IBU	Ibuprofen
NSAIDs	Non-steroidal anti-inflammatory drugs
