# Supplementary material for: Geopolymer-Based Materials for the Removal of Ibuprofen: A Preliminary Study
Source: Molecules. 2024 May 8;29(10):2210. doi: 10.3390/molecules29102210 (PMC11124334; doi:10.3390/molecules29102210)
Supplement: Supplementary file 1 [file molecules-29-02210-s001.zip › molecules-2972094-supplementary.pdf]

Supplementary material

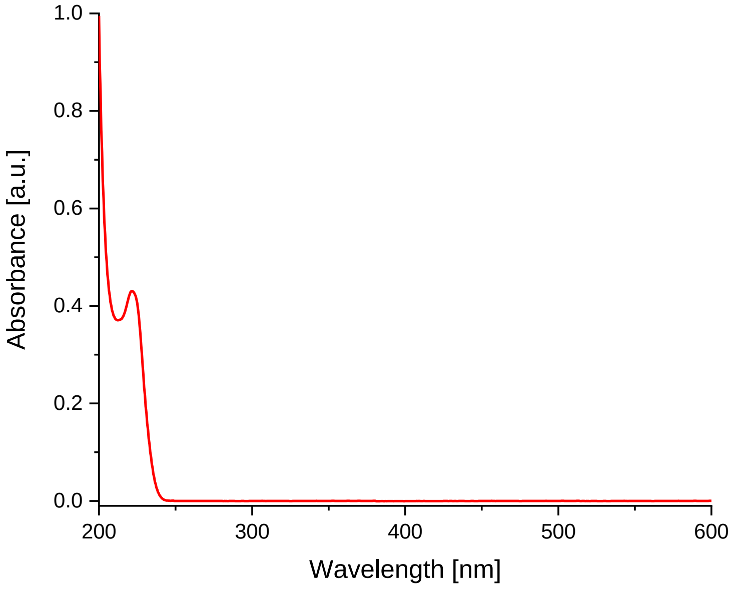

Figure S1. UV-Vis spectrum of ibuprofen with a concentration of 10 mg/L recorded from 200 to 600 range of wavelength.

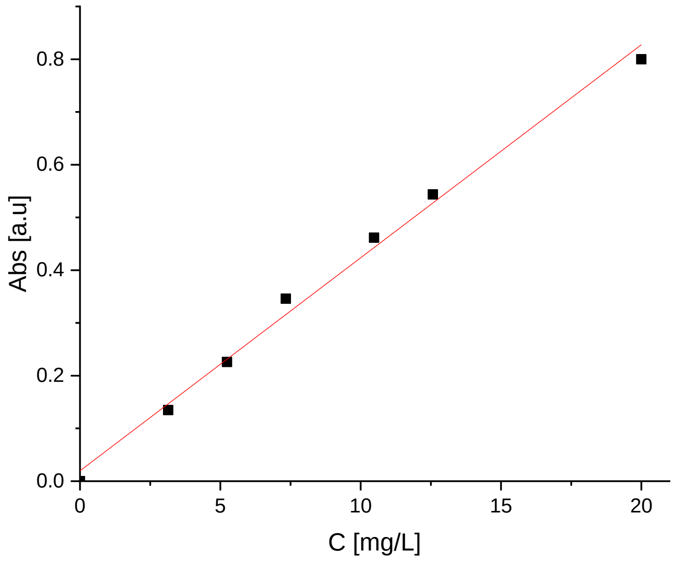

Figure S2. UV-VIS calibration curve for ibuprofen (IBU) solutions in water.
